# The Evaluation of Single-Sided Total Knee Arthroplasty Versus Simultaneous Bilateral Total Knee Arthroplasty Improvements and Postoperative Progression Based on Patient-Based Outcome Scoring: A Rural Retrospective Clinical Orthopaedic Study

**DOI:** 10.5435/JAAOSGlobal-D-19-00069

**Published:** 2019-07-09

**Authors:** Arielle Harnik, Jay Boughanem, Patrick Hart, Omer Margolin, Landon Collins, Ryan Hilton

**Affiliations:** From Division of Surgery, Hilo Medical Center (Ms. Harnik, Dr. Boughanem, Mr. Margolin, Mr. Collins, and Mr. Hilton); Hilo Bone and Joint Clinic (Ms. Harnik, Dr. Boughanem, Mr. Margolin, Mr. Collins, and Mr. Hilton); and University of Hawaii at Hilo, Hilo, HI (Dr. Hart).

## Abstract

**Introduction:**

Both graduated single-sided total knee arthroplasty (SSTKA) and simultaneous bilateral total knee arthroplasty (SBTKA) are viable options for bilateral knee arthritis, and deciding which option to pursue is still debated. We aim to compare the two modalities using the patient-based oxford knee score and Visual analog pain scores in micropolitan settings.

**Methods:**

Oxford knee score and Visual analog pain scores were administered preoperatively and postoperatively 1, 6, and 12 months to 115 patients who underwent total knee arthroplasty. The 115 cohort was divided into two groups, those who underwent SSTKA and those who received SBTKA.

**Results:**

Cross-group analysis showed a significant difference with oxford scores at the 1-month postoperative interval (*P* = 0.026). The within-group analysis of the delta oxford knee scores displayed postoperative improvement at the 0.05 level of significance at 1, 6, and 12 months.

**Discussion:**

This study indicates that the patient-based outcome measures for the SBTKA group lagged behind the SSTKA group. The overall improvement a year out from surgery is comparable, and both groups had significant improvement in function. The SBTKA patient group had markedly lower functional outcome measures based on oxford scores at 1 month post-op compared with the SSTKA group; this may help in decision-making and patient selection.

## Introduction

Total knee arthroplasty (TKA) has been the mainstay of treatment of severe arthritis of the knee since the late 1900s in the United States.^3,12^ Knee arthritis is the major cause of disability in older adults (65 to 80 years old).^31^ In addition, the future occurrence of osteoarthritis is estimated to increase 40% by the year 2025.^19^ In previous decades, simultaneous bilateral TKA (SBTKA) has been performed on select patients who suffer from bilateral knee arthritis. It is shown that in 2007 there were 611,000 TKAs that took place in the United States, 7% were SBTKA(s).^1,3,13,19^ For older adult patients who suffer from bilateral knee arthritis, there are no specific guidelines that can assist the clinician through the decision whether to perform a staged single-sided TKA (SSTKA) (also categorized as a staged bilateral TKA or unilateral TKA) or SBTKA.^1,3,4,14,29,31^ SBTKA does have some considerable benefits for patients such as a less postoperative hospital stay and a more cost-effective procedure compared with staged SSTKA.^1,7,27,28^ However, patients who underwent SBTKA showed notable increase in mortality and morbidity rates and higher risks for cardiovascular and neurologic complications.^1,8,10,11,29,30,35^ Most of these postoperative TKA complication data are derived from meta-analyses conducted in urban areas. However, in a long-term measure study comparing complication rates within micropolitan areas and metropolitan areas that received successful TKAs, the mortality rates and perisurgical complication rates were not markedly different.^32^

In the last decade, patient-based outcome studies have been more prevalent and the emphasis on patient satisfaction has taken greater significance.^2,15,23^ The experience of the patient can provide further insight for the clinician and other patients who may be considering those types of knee replacement procedures.^16–18^ In addition, patient-based questionnaires have been proven to hold validity and reliability.^2,5,6,20,21^ There are a variety of validated patient-based outcome scores to evaluate the function and progression of knee replacements. The Western Ontario and McMaster Universities Osteoarthritis scale developed in 1982 and the Knee Society Score developed in 1989^14^ are two such examples that are popular in North American orthopaedic clinical trials.^6,9,19^ Currently, there are few studies in the United States that assess SSTKA and SBTKA patient groups in comparison to their preoperative and postoperative oxford knee score (OKS). Utilization of OKS is even less prominently used in relation with healthcare communities within micropolitan areas that are considered rural.^33^ This could be because of the oxford questionnaire being more prevalent in the United Kingdom with most of its utilization in orthopaedic studies in metropolitan areas.^6,9^

The oxford questionnaires (which include the oxford knee scores and oxford hip score) were established and used in 1996 and 1998, respectively, and have been the main patient-based outcome assessments within many studies regarding joint-specific replacements for the past 20 years in the United Kingdom.^21^ It was determined that the oxford scoring system is the best and most reliable scoring system for patient-based assessments of joint-specific replacements that cannot be influenced by other comorbidities, in comparison to the other scoring systems for this category.^6,21,22^ It has also been apparent that one of the most important factors that affect the outcome after a joint replacement procedure is the preoperative oxford score that the patient provides before surgery.^24^ To analyze the change or progression of oxford scores, pre-oxford and post-oxford scores must be obtained, especially if the cohort of patients under study has undergone different treatments or surgical joint replacement procedures.^21^ After any joint replacement, most improvements in function and oxford scores can be observed within the first year.^2,21,25^

Studies on analog pain scores have also shown insights into patient-based pain levels of SSTKA versus SBTKA. One study measured the average analog pain scores from SSTKA versus SBTKA days after their knee replacement surgery, averaging the pain scores from days 1 to 3 postoperatively. They found that the SSTKA group had a markedly lower average pain than the SBTKA replacements on day 1 after surgery.^26^

The purpose of this study is to retrospectively investigate patient pain levels and oxford knee scores of a cohort that successfully underwent either SSTKA or SBTKA knee replacements within a micropolitan setting to determine which knee replacement procedure is the best choice for reducing a patient's postoperative recovery time and increase patient satisfaction. The OKS was chosen as a robust joint-specific test to evaluate subjective patient postoperative knee function improvement. Instead of focusing on short-term pain analysis for both groups after knee replacement,^26^ we measured average pain level scores at 1, 6, and 12 months to compare outcomes from SSTKA and SBTKA groups. First, we hypothesize that the SSTKA group at 1 and 6 months will have better average delta pain and average delta oxford knee scores in comparison to the SBTKA group at 1 and 6 months. Second, as stated in previous studies,^2,21,25^ we also postulate that by the 12-month follow-up, both groups will show equivalent delta pain and oxford score improvements.

## Methods

A total of 124 orthopaedic knee procedures were performed between 2016 and 2017 in our rural institution orthopaedic division. The exact start and end dates for this retrospective chart analysis study was from January 1, 2016, to December 31, 2017. All patients in this study underwent TKA due to a form of arthritis in the knee(s). The cohort was divided into two groups; there were a total of 112 patients who received SSTKAs and a total of 12 patients who received SBTKAs. For the 12 patients who underwent SBTKA, each knee was recorded as an independent case providing 24 procedures for this group. A random generator was used to indiscriminately select between left and right knees from each patient to eliminate individual biases for our statistical analyses. Exclusion criteria provided a cohort total of 115 total knee cases. Exclusion criteria included those patients who declined filling in the patient-based questionnaires during their clinical follow-ups after receiving either of these knee procedures. For the patient exclusion category that did not fill-out the oxford questionnaire, there were a total of nine patients removed from the SSTKA group. In summary, we had a total of 103 total knee procedure(s) in the SSTKA group and a total of 12 knee procedure(s) in the SBTKA group. The inclusion criteria included preoperative and postoperative patients who had osteoarthritis, rheumatoid arthritis, trauma, infection, and cardiovascular or neurologic disorders. Postoperative revisions were also included and recorded. In the SSTKA patient group, three patient(s) returned to the operating room, all three were recorded as having stiffness treated with arthroscopic débridement and manipulation under anesthesia. In the SBTKA group, one patient returned to the operating room because of one-sided wound dehiscence and stiffness, manipulation under anesthesia recorded. There were no patient demographic characteristic cutoffs' or selection bias for patient's sex, body mass index (BMI), or age. The average age of patients within the SSTKA group was 70.7 ± 8.31, and the average age of patients within the SBTKA group was 72.2 ± 7.27. The average BMI in the SSTKA group was 30.3 ± 5.65 kg/m^2^ and in the SBTKA group the average BMI was 28.5 ± 2.85 kg/m^2^. There were 43% male and 57% female patients analyzed within this study, and they were primarily osteoarthritic. A cross-group analysis using a Mann-Whitney test was used to verify that there was no notable difference between patient demographic characteristics and preoperative or postoperative scores at 1, 6, and 12 months to eliminate selection bias (Tables 1–3). Missing data were also recorded along with oxford knee and pain level score averages. No preoperative oxford knee scores (SSTKA n = 103 and SBTKA n = 12) and pain level scores (SSTKA n = 103 and SBTKA n = 12) were missing from both TKA groups. For the postoperative oxford knee scores in the SSTKA group, at month 1 (n = 92), 11 of 103 patients had missing oxford scores with zero missing pain level scores (n = 103), at month 6 (n = 100), there were three patients of 103 had missing oxford scores and zero missing pain level scores (n = 103), at month 12 (n = 102), one patient of 103 had missing oxford score and zero missing pain level scores (n = 103). For the postoperative oxford knee scores in the SBTKA group at month 1 (n = 11) only 1 patient of 12 had a missing oxford score and zero missing pain level scores (n = 12) (Tables 1–3). Patients with missing oxford scores were excluded in the data analysis that included delta oxford scores; however, their pain level scores were included in the data analysis of delta pain levels. Patient follow-ups after surgery took place at a micropolitan orthopaedic out-patient clinic and scores were retrospectively extracted at 1, 6, and 12 months postsurgery. The follow-ups consisted of a physical examination, radiographs, and examination of pain and possible complications. Level of pain and progress of each patient's replacement was evaluated preoperatively and postoperatively by the patient from their clinical follow-up OKS, part of their standard medical records. Pre-evaluation mean and range of OKS and pain level scores are also shown in (Table 1). All preoperative and postoperative data from the patient's clinical examinations and radiographs were recorded in the electronic medical record (EMR) system. The OKS, pain level, and percent improvement at the preoperative and 6 month's postoperative time intervals were originally collected from the local EMR system. For all postoperative clinical visits, patients were asked to complete an oxford questionnaire, rate their pain and percent improvement, and their responses were uploaded onto the EMR system.

**Table 1:**
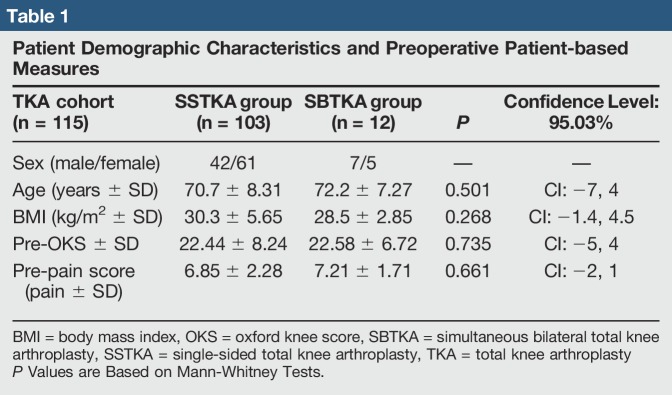
Patient Demographic Characteristics and Preoperative Patient-based Measures

**Table 2:**
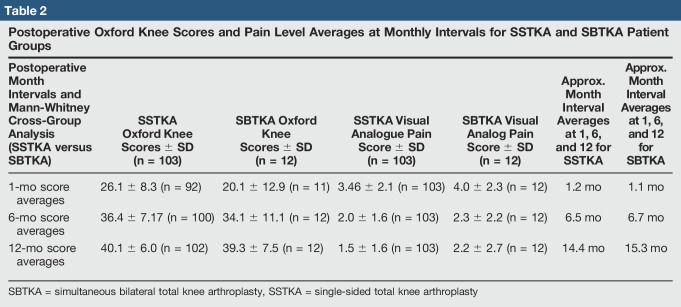
Postoperative Oxford Knee Scores and Pain Level Averages at Monthly Intervals for SSTKA and SBTKA Patient Groups

**Table 3:**
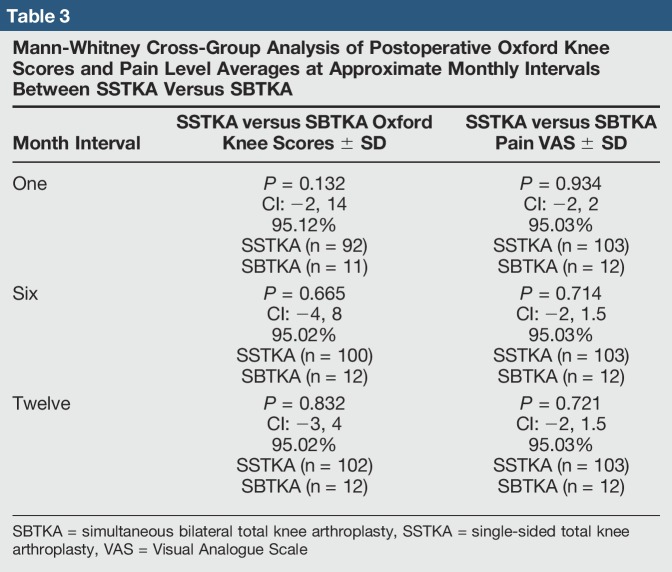
Mann-Whitney Cross-Group Analysis of Postoperative Oxford Knee Scores and Pain Level Averages at Approximate Monthly Intervals Between SSTKA Versus SBTKA

All TKA procedures in both groups were performed by the same orthopaedic surgeon at a micropolitan district hospital with updated medical instruments. Before the surgery, the decision to proceed with SSTKA versus SBTKA procedure was made between the patient and surgeon based on the patient's age, comorbidities, and procedure preference. Patients were made aware of the expected beneficial outcomes and potential risks with surgery.

The OKS was used during preoperative and postoperative evaluation of the patient. With this questionnaire, patients were able to rank the pain and function of their knee replacement during each clinical follow-up. It is a questionnaire that consists of 12 questions that pertains to the mobility, psychological fear, and pain sensitivity of their current knee condition before and after the surgery. Each question was a multiple choice, with responses ranked from 1 to 4, with the overall sum total of the best patient-based oxford knee scores equaling 48. A patient who gives a higher sum oxford score in comparison to their preoperative oxford score indicates improvement with their knee replacement.

The Visual Analogue Scale was also used along with the preoperative and postoperative OKS evaluation. Using the Visual Analogue Scale with a pain score from 0 to 10, the patients were able to rank their average knee pain that they had within the past month postsurgery, with 0 being no pain and 10 being the worst pain.

### Statistical Analysis Methods

**Figure 1:**
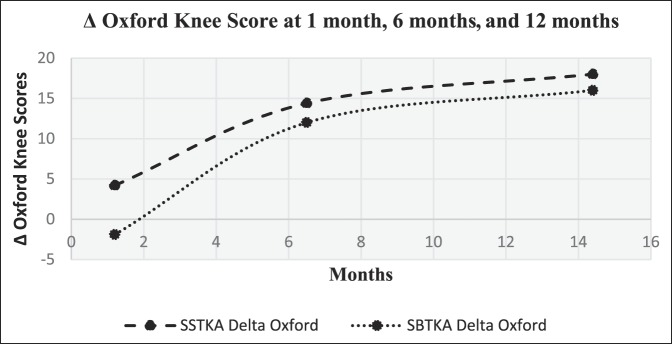
Graph showing patient-based delta oxford score averages from SSTKA and SBTKA groups at 1, 6, and 12 months. SBTKA = simultaneous bilateral total knee arthroplasty, SSTKA = single-sided total knee arthroplasty

Statistical tests were conducted on Minitab software version 18.0. We used one-sided Mann-Whitney tests with an alpha-level of 0.05 to do cross-group analyses with the delta oxford scores and pain levels from each patient group at the same time intervals. In addition, we used one-sided paired *t*-tests to analyze score progression within each group. We hypothesized that the median delta oxford knee scores of the SSTKA group at 1 and 6 months would be higher than that of the SBTKA group and that the median delta pain score of the SSTKA group at 1 and 6 months would be lower than that of the SBTKA group. A one-sided Mann-Whitney test was used to determine whether the median delta pain level of the SSTKA group (n_1_ = 103) at 1 month, 6 months, and 12 months was markedly less than the median delta pain level of the SBTKA group (n_2_ = 12) at the same time intervals (Table 4.). We also used a one-sided Mann-Whitney test to determine whether the median delta oxford knee scores of the SSTKA group at 1 month (n_1_ = 92), 6 months (n_1_ = 100), and 12 months (n_1_ = 102) was markedly greater than the median delta oxford knee scores of the SBTKA group at the same time intervals (Figure 1); 1 month (n_2_ = 11), 6 months (n_2_ = 12), and 12 months (n_2_ = 12) (Table 5).

**Table 4:**
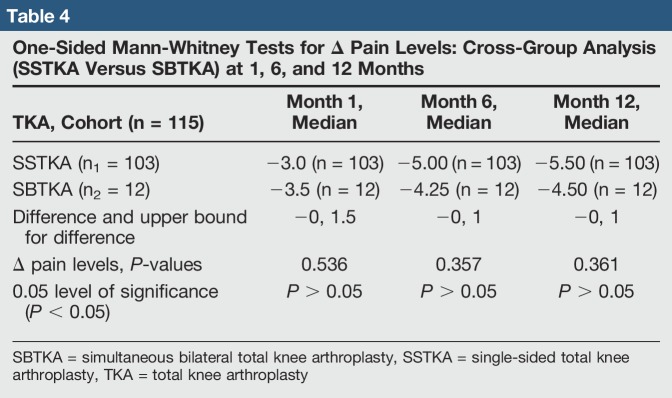
One-Sided Mann-Whitney Tests for Δ Pain Levels: Cross-Group Analysis (SSTKA Versus SBTKA) at 1, 6, and 12 Months

**Table 5:**
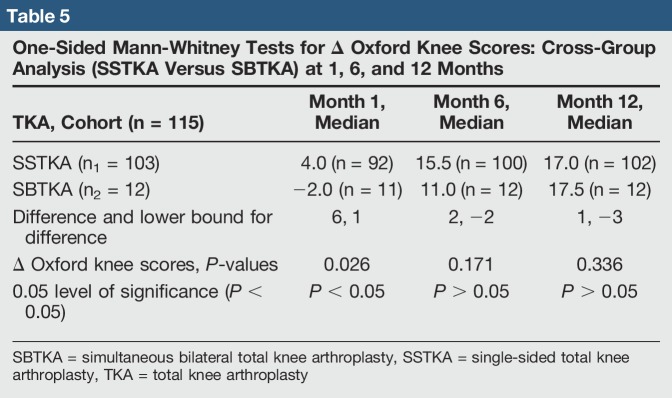
One-Sided Mann-Whitney Tests for Δ Oxford Knee Scores: Cross-Group Analysis (SSTKA Versus SBTKA) at 1, 6, and 12 Months

We then conducted a one-sided paired *t*-test to determine whether there was any notable improvement over time (1 to 12 months) with the patient-based delta oxford knee and pain scores within each patient group(s). Specifically, we tested whether the SSTKA group's mean delta pain level at 12 months was markedly less than 6 months or 1 month (Table 6.). This test was also done to analyze SSTKA progression for the delta oxford knee scores to determine whether the delta oxford scores were markedly greater at 12 (n = 102) months than at 6 months (n = 100) or 1 month (n = 92). The same steps were taken with the delta pain and oxford knee scores progression at the postoperative time intervals with the SBTKA group using a one-sided paired *t*-test (Tables 7 and 8).

**Table 6:**
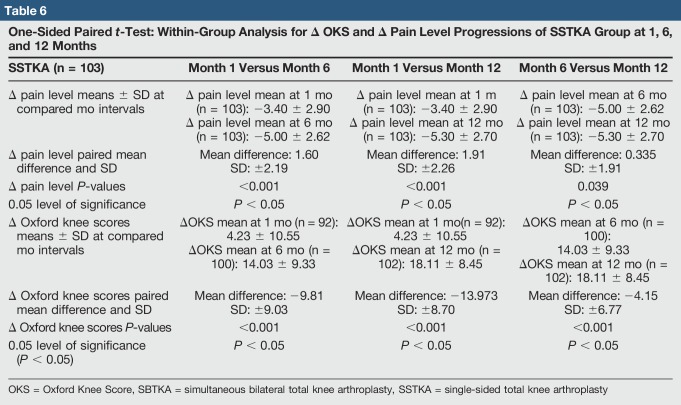
One-Sided Paired *t*-Test: Within-Group Analysis for Δ OKS and Δ Pain Level Progressions of SSTKA Group at 1, 6, and 12 Months

**Table 7:**
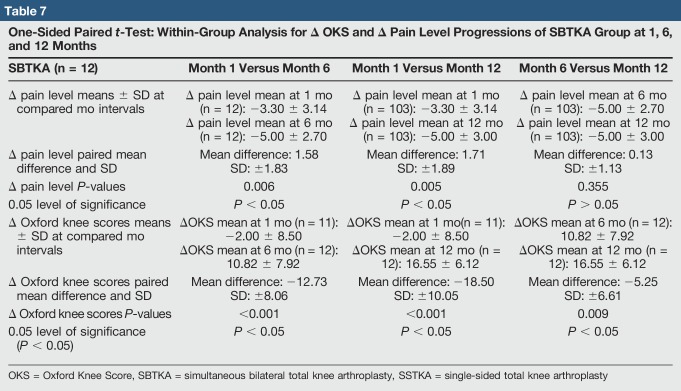
One-Sided Paired *t*-Test: Within-Group Analysis for Δ OKS and Δ Pain Level Progressions of SBTKA Group at 1, 6, and 12 Months

**Table 8:**
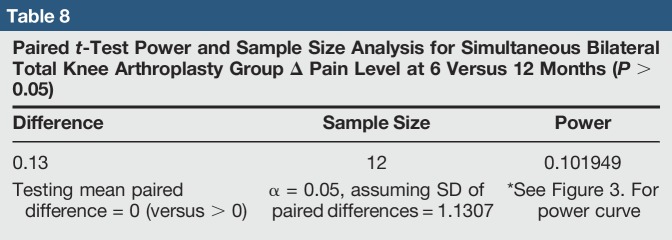
Paired *t*-Test Power and Sample Size Analysis for Simultaneous Bilateral Total Knee Arthroplasty Group Δ Pain Level at 6 Versus 12 Months (*P* > 0.05)

## Results

**Figure 2:**
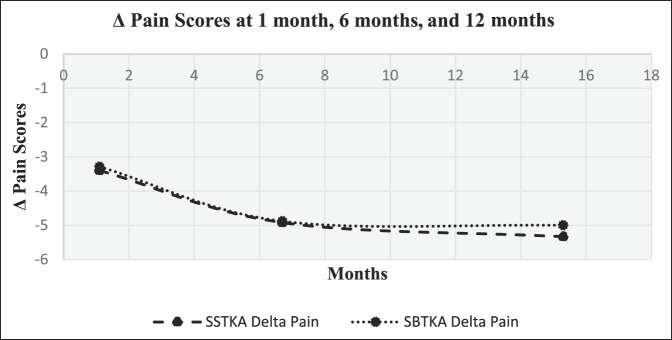
Graph showing patient-based delta pain score averages from SSTKA and SBTKA groups at 1, 6, and 12 months. SBTKA = simultaneous bilateral total knee arthroplasty, SSTKA = single-sided total knee arthroplasty

The follow-up time for both groups in this study was ≥1 year. Delta OKSs and delta pain scores that were collected and recorded from each group at 1, 6, and 12 months postoperatively were used to create time series plot diagrams. An increasing trend was noted for the delta oxford scores versus time and a decreasing trend of delta pain levels versus time for both groups. Delta oxford scores and pain level average score progression for SSTKA and SBTKA groups at 1, 6, and 12 months can be seen in (Figures 1 and 2).

**Figure 3:**
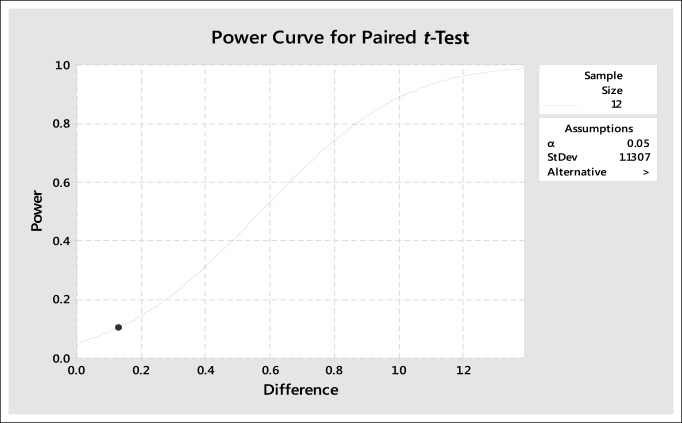
Graph showing one-sided paired *t*-test power curve from within group Δ pain level analysis at 6 versus 12 months for the SBTKA group (*P* > 0.05). SBTKA = simultaneous bilateral total knee arthroplasty

There was no significant difference in median delta pain scores between the two groups at 1 month (*P* = 0.648, Confidence Level (CL) = 95.07%), 6 months (*P* = 0.357, CL = 95.07%), and 12 months (*P* = 0.361, CL = 95.07%) (Table 4). There was a significant difference in the median delta oxford scores between the two groups at 1 month (*P* = 0.026, CL = 95.05%) but no significant difference at 6 months (*P* = 0.171, CL = 95.06%) and 12 months (*P* = 0.336, CL = 95.03%) (Table 5). In the SSTKA group, a significant difference was noted in delta pain level score progression at 1 versus 6 months (*P* ≤ 0.001, 90% Confidence Interval (CI) = 1.2139, 1.9317), 1 versus 12 months (*P* ≤ 0.001, 90% CI = 1.5380, 2.2775), and 6 versus 12 months (*P* = 0.039, 90% CI = 0.022729, 0.64717). In the SSTKA group analysis, there was a significant difference in delta oxford knee scores progression at 1 versus 6 months (*P* ≤ 0.001, 90% CI = −11.389, −8.2223), 1 versus 12 months (*P* ≤ 0.001, 90% CI = −15.487, −12.458), and 6 versus 12 months (*P* ≤ 0.001, 90% CI = −5.2815, −3.0215) (Table 6) Regarding delta pain level score progression in the SBTKA group analysis, a significant difference was observed at 1 versus 6 months (*P* = 0.006, 90% CI = 0.63360, 2.53331) and 1 versus 12 months (*P* = 0.005, 90% CI = 0.72930, 2.6874). At 6 versus 12 months (*P* = 0.355, 90% CI = −0.46117, 0.71117) no significant difference was observed (Table 7). A paired *t*-test power analysis showed a low power (10%) to detect significance (Table 8 and Figure 3). In the SBTKA group, there was also a significant difference in delta oxford knee scores progression at 1 versus 6 months (*P* ≤ 0.001, 90% CI = −17,134, −8.3208), 1 versus 12 months (*P* ≤ 0.001, 90% CI = −23.950, −12.961), and 6 versus 12 months (*P* = 0.009, 90% CI = −8.6755, −1.8245) (Table 7).

## Discussion

This study retrospectively analyzes the progress of SSTKA and SBTKA patient groups for a period of 1 year after knee replacement to compare functionality and recovery using patient-based OKSs and pain level scores for patients from a micropolitan community. Currently, there is no standard of care to help determine which total knee procedure would have the best long-term outcome for elderly patients with severe knee arthritis.

Our hypotheses were partially validated. First, we hypothesize that the SSTKA group at 1 and 6 months will have better average delta pain and oxford knee scores in comparison to the SBTKA group at 1 and 6 months. There was a significant difference (*P* < 0.05) between the SSTKA group and the SBTKA group with 12.5% higher overall functional level in the SSTKA group at 1 month. However, the same hypothesis test at postoperative 6 and 12 months between the two total knee groups displayed no notable difference (Table 5). The cross-group analysis also showed that no significant difference in pain was observed at the same time intervals (Table 4). This outcome partially invalidates our first hypothesis.

A significant improvement was observed in functional level (*P* < 0.05) within both groups at all intervals (1 to 6, 1 to 12, and 6 to 12 months). In addition, significant pain improvement was observed in the SSTKA group (*P* < 0.05) at all intervals (Table 6). However, the SBTKA group did not express significant improvement in average delta pain levels after 6 months (*P* = 0.355), postoperatively (Table 7). This partially validates our second hypothesis.

Other studies that incorporated patient-based questionnaires to evaluate SBTKA and SSTKA postoperative functionality levels ≥1 year out had similar outcomes. In an orthopaedic study published in 2009 that compared SBTKA and SSTKA patient recipients with a healthy control group for 2 years showed notable functional improvement in both groups.^34^ Another study published in 2015 used the Knee Injury and Osteoarthritis Outcome Score to evaluate post-op functional capabilities for both TKA groups. Both unilateral and simultaneous bilateral patient groups showed notable functional improvements versus time. However, they did not find any notable difference in functionality when comparing scores between the two groups versus time.^36^

There were limitations for this study; one of the main ones being the lack of statistical power. The retrospective nature of our study did not accumulate a large number of patients, providing uneven sample sizes for the SSTKA and SBTKA groups. Possible factors that contribute to the lack of power are the low volume rate of SBTKA per year at our rural hospital.^32,37^ Although we had a low power for this study, our statistical testing did detect a significant difference with postoperative knee functionality between the two groups at 1 month; this infers there is a difference. There are also limited rural and metropolitan studies that evaluate pain progression of SBTKA patient recipients in comparison to SSTKA patients after 6-month post-op periods. In addition, there are also limited studies that explore functionality levels between TKA groups 3 to 6 weeks postoperatively.

Our results indicate that overall function (based on OKS) of the SBTKA group lag behind the SSTKA group at 1 month postoperatively. This is based on the 12.5% lower functional level in the SBTKA group versus the SSTKA group at 1 month postoperative (*P* < 0.05). These findings indicate that SBTKA patients might have decreased short-term satisfaction after surgery compared with the SSTKA patients. The markedly lower functional level in the SBTKA group in the early (1 month) postoperative period may help the clinician in SBTKA patient selection. In our institution, we now encourage patients with bilateral degenerative joint disease in the knee(s) who have inadequate collateral social support^38^ to undergo single-sided staged TKA.
